# Transient abnormal myelopoiesis with pericardial effusion in Down syndrome: Case report

**DOI:** 10.1002/ccr3.2184

**Published:** 2019-05-17

**Authors:** Bianca Francisco Falasco, Brenda Durante, Daniel Kanaan Faria, Caroline Silvério Faria, Débora Cristina Batista Rosolen, Leila Antonangelo

**Affiliations:** ^1^ Divisao de Patologia Clinica ‐ Departamento de Patologia, Hospital das Clinicas HCFMUSP, Faculdade de Medicina Universidade de Sao Paulo Sao Paulo SP Brazil; ^2^ Laboratorio de Investigacao Medica (LIM 03), Faculdade de Medicina FMUSP Universidade de Sao Paulo Sao Paulo SP Brazil

**Keywords:** basophils, Down syndrome, eosinophils, pericardial effusion, transient abnormal myelopoiesis

## Abstract

Pericardial effusion associated with transient abnormal myelopoiesis in Down's syndrome neonates needs to be diagnosed in a timely manner, and the comorbidities must be treated to prevent mortality. To our knowledge, the occurrence of basophilic/eosinophilic pericardial effusion without an increase of these cells in the peripheral blood and with no evidence of associated hypothyroidism is rare.

## 1 INTRODUCTION

Down syndrome (DS) patients can develop transient abnormal myelopoiesis, characterized by leukocytosis, circulating blasts, and thrombocytopenia. Pericardial effusion is an important cause of morbidity. We reported DS/TAM neonate with eosinophilic/basophilic pericardial effusion that did not present these cells at peripheral blood. To date, it is rare form of disease presentation.

Down syndrome, or trisomy 21, is one of the most frequent chromosomal abnormalities among neonates. The presence of an extra copy of the chromosome causes an imbalance in the genic expression, resulting in the phenotypic characteristics of patients with DS and several changes in development. The incidence of DS is influenced by the increase in maternal age, with a worldwide frequency of approximately 1:1000 births.^1^ Of these, approximately 50% have congenital heart disease^1^ and a high risk for hematological clonal diseases, such as transient abnormal myelopoiesis (TAM) and myeloid leukemia.^2^

Transient abnormal myelopoiesis develops in approximately 10%‐15% of patients with DS at birth or in the first days of life and usually resolves spontaneously within 3 months; however, in some cases, myeloid leukemia can manifest later.^2^, ^3^, ^4^ The proliferation of immature cells is of megakaryoblastic origin, and the cells accumulate in the liver, bone marrow, and peripheral blood (PB).^4^, ^5^ Cavity effusions with or without generalized edema are usually observed in fetuses with DS associated with TAM and/or hypothyroidism, which are also common in these patients.^2^, ^5^, ^6^

The pathophysiology of TAM involves a number of factors, including the proliferation of abnormal hematopoietic precursors in the fetal liver caused by genetic instability in the presence of trisomy 21 and by the acquisition of mutations in the GATA1 gene, located on chromosome X, which is essential for erythroid and megakaryocytic maturation.^3^, ^4^, ^7^ Leukemic evolution, however, requires the acquisition of additional mutations.^2^, ^4^ Laboratory findings include PB leukocytosis, basophilia, and thrombocytopenia, and the presence of blasts, which generally express immature surface and myeloid antigens (CD34, CD117 and CD33, CD13, respectively), and at least one platelet antigen (CD36, CD41a, CD41b, or CD61); there may also be aberrant expression of CD56 and CD7.^2^, ^3^ Although most cases have a spontaneous resolution not requiring specific treatment, patients with unfavorable evolution must be treated with chemotherapy in low doses, with a reduction in mortality.^3^

We described a newborn with DS, TAM, and basophilic/eosinophilic pericardial effusion (PE) who did not present with an increase in these cells in the PB and with no evidence of associated hypothyroidism.

## 2 CASE REPORT

A 14‐day‐old male newborn was admitted to the Emergency Room of the Hospital das Clinicas da Universidade de Sao Paulo in regular clinical condition, coming from another hospital unit where the delivery occurred. The postnatal PB showed significant leukocytosis (57 900/mm^3^) with blasts. Due to his clinical condition, he was transferred to the neonatal intensive care unit. He evolved on the 2nd day of birth with hypothermia (34.5°C), jaundice, and persistence of leukocytosis with blasts. In the hypothesis of sepsis, antibiotic therapy (ampicillin and gentamicin) was started for four days. The mother was 22 years old, with two previous pregnancies and no history of abortions and consanguinity.

In the physical examination, the pediatrician observed typical DS facies, a simian fold on hands, the presence of diffuse nodular erythema and mild hypotonicity, with no cardiovascular and/or respiratory disorders. However, on the third day of life, the patient developed respiratory distress, O_2_ desaturation, and acute renal failure, requiring orotracheal intubation and vasoactive drug use.

**Figure 1:**
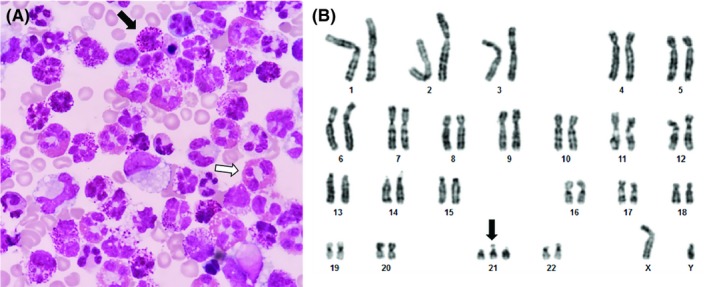
A, Cytological examination of the pericardial fluid showing a large number of basophils (black arrow) and eosinophils (white arrow); Leishman staining, 630×; B, Patient's karyotype showing the trisomy of chromosome 21 (black arrow)

The echocardiogram detected cardiac tamponade with restriction on biventricular filling; pericardiocentesis was performed by Marfan puncture, with withdrawal of 21 mL of pericardial fluid (PF), whose laboratorial analysis showed proteins 2.9 g/dL, lactic dehydrogenase 413 U/L, and glucose 101 mg/dL; cellularity was 1200/mm^3^, with 82% leukocytes (55% basophils and 22% eosinophils; Figure 1A). Pericardial fluid immunophenotyping by flow cytometry did not reveal blasts in the sample. The patient's karyotype confirmed Down syndrome (47,XY,+21; Figure 1B). The pediatrician suggested the diagnosis of DS associated with TAM and initiated low‐dose chemotherapy (cytarabine) for five complete cycles. The patient presented with tumor lysis syndrome, ascites, and pleural effusion during hospitalization. Because of the worsening of patient evolution, the pediatrician made the diagnostic hypothesis of fungal sepsis, although fungi did not grow in culture; the pediatrician opted for the introduction of micafungin (10 days). The patient evolved with gradual improvement and was discharged from the hospital on the 32nd day. The results of the main laboratory and image tests are described in Table 1.

**Table 1:** Laboratory and image tests per day of hospitalization

Day	Laboratory tests		Image tests
1st	Leukocytes: 66 950 cells/mm^3^ Platelets: 103 000/mm^3^	Partial biotinidase deficiency	Free fluid in abdominal cavity; discrete pyelocalyceal dilatation on the right
2nd	PF cytology: Nucleated cells: 984 000 cells/mm^3^ [Neut: 15%, Eos: 22%, Baso: 55%, Lymph: 6%, Mono: 2%], Macrophages (12%), Mesothelial cells (6%); PF immunophenotyping: absence of blasts, large quantity of basophils		Patent foramen ovale with left‐right atrial flow, PE—signs of restriction to ventricular filling; Marfan puncture (21 mL)
4th	PB: 23% cells CD45+/++, CD34+, CD117+, HLA‐DR+, CD33+, CD7+ and CD56+, precursor cells in PB without antigen‐specific expression of lineage suggestive of TAM associated with DS	Uric acid: 7.9 mg/dL	Patent foramen ovale with left‐right atrial flow; absence of PE; left ventricular systolic dysfunction after reduction in vasoactive drugs
5th	Leuko: 44 350 cells/mm^3^ with 29% immature cells; Plat: 95 000/mm^3^; PT: INR: 1.42; Karyotype: 47,XY,+21	Uric acid: 9.4 mg/dL	Not performed
6th	Hb: 12 g/dL; Ht: 31%; Leuko: 7040 cells/mm^3^ with 9% immature cells; Plat: 83 000/mm^3^ APTT: R = 1.63; PT: INR = 1.19	Uric acid: 9.5 mg/dL	
7th	Hb: 10.9 g/dL; Ht: 29.1%; Leuko: 5440 cells/mm^3^ with no immature cells; Plat: 111 000/mm^3^	Uric acid: 0.6 mg/dL CRP: 30.91 mg/L	
9th	Hb: 9.6 g/dL; Ht: 27.2%; Leuko: 2640 cells/mm^3^ with no immature cells; Plat: 76 000/mm^3^; APTT: R = 1.59; PT: INR = 1.04	Uric acid: 2.0 mg/dL	
11th	Hb: 13.4 g/dL; Ht: 38.4%; Leuko: 4940 cells/mm^3^ with no immature cells; Plat: 30 000/mm^3^	CRP: 40.59 mg/L	
15th	Hb: 12.8 g/dL; Ht: 37.4%; Leuko: 4260 mil cells/mm^3^ with no immature cells; Plat: 130 000/mm^3^; APTT: R = 1.11; PT: INR = 1.04	CRP: 122.54 mg/L	Patent foramen ovale with left‐right atrial flow; absence of PE; preserved left ventricular function
23th	Hb: 14.2 g/dL; Ht: 41%; Leuko: 11 850 cells/mm^3^ with no immature cells; Plat: 289 000/mm^3^	CRP: 5.1 mg/L	

Abbreviations: APTT, Activated Partial Thromboplastin Time; Baso, Basophils; CRP, C‐reactive protein; DS, Down syndrome; Eos, Eosinophils; Hb, Hemoglobin; Ht, Hematocrit; INR, International Normalized Ratio; Leuko, Leukocytes; Lymph, Lymphocytes; Mono, Monocytes; Neut, Neutrophils; PB, Peripheral Blood; PE, Pericardial Effusion; PF, Pericardial fluid; Plat, Platelets; PT, Prothrombin Time; R, Ratio; TAM, Transient Abnormal Myelopoiesis.

## 3 DISCUSSION

Patients with TAM who evolve with PE generally present increased morbidity, especially if associated with hydropsy, heart failure, and hypothyroidism.^5^, ^6^ There are few reports in the literature of TAM associated with cavity effusions, and to date, no described case presents an increased number of basophils and eosinophils in the pericardial fluid without an increase in these cells in the PB. We highlight that the main abnormalities observed in the PB were the presence of blast cells and thrombocytopenia.

The blast cells involved in TAM are immature megakaryocytes or common erythroid‐megakaryocytic precursors that are morphologically indistinguishable from those found in DS‐associated myeloid leukemia.^2^ However, Suda et al^8^ reported that, when submitted to different colony stimulating factors in vitro, approximately 50% of blasts from patients with trisomy 21 were able to proliferate and differentiate into basophils, eosinophils, erythrocytes, macrophages, and neutrophils, demonstrating the persistent ability of these cells to differentiate and the transient aspect of abnormal myelopoiesis.

In Table 2, we summarize the literature reports of patients with DS‐ and TAM‐associated PE. From the reported cases, three cases presented an increased number of eosinophils in the PE,^5^, ^9^, ^10^ and in two cases, there are no mentions of the cytological aspects of PE.^6^, ^11^

**Table 2:** Literature cases of DS‐TAM and main clinical findings

Author/Year	Country	Age/Gender	Clinical findings	Main hematological findings	Karyotype	Pericardial effusion cytology	Diagnosis	Treatment	Follow‐up
Kusanagi et al 1998^9^	Japan	At birth, female	Hepatosplenomegaly, ventricular septal thickening, PE, hydropsy	Leukocytes: 104 000 cells/mm^3^; 27.5% blasts, and 28% eosinophils	47,XX,+21	Large number of eosinophils	DS, Hypereosinophilic syndrome and TAM	Prednisolone	Normalized at 8 wk, with normal PB and PE resolution
Hirashima et al 2000^6^	Japan	At birth, male	Ventricular septal defect, PE	Leukocytes: 36 100 cells/mm^3^; 49% blasts	47,XY,+21	Not mentioned	DS and TAM	Prednisolone, L‐thyroxine	Normalized in 127 d without blasts
Shenoy et al 2008^5^	India	14 d, male	Progressive respiratory distress, cardiomegaly, hepatosplenomegaly atrial septal defect and PE	Leukocytes: 150 000 cells/mm^3^; 46% blasts	Not mentioned	29% of eosinophils	DS and TAM	Prednisolone	Normalized in 3 wk with no blasts in PB and PE resolution
Gosavi et al 2011^11^	India	At birth, male	Hepatosplenomegaly, ascites, PE	Leukocytes: 56 000 cells/mm^3^; 44% blasts, basophilia	47,XY,+21	Not mentioned	DS and TAM	Not mentioned	Normalized in 2 mo, but with 7% of basophils in PB
Shitara et al 2017^10^	Japan	At birth, female	Hepatomegaly, cardiomegaly, PE, pulmonary hypertension	Leukocytes: 36 800 cells/mm^3^; 19% blasts and 32% eosinophils	47,XX,+21	41% of eosinophils	DS and TAM	Prednisolone	Normalized at 49 d, decreasing of blasts and eosinophils in PB and, PE resolution

Abbreviations: DS, Down syndrome; PB, Peripheral Blood; PE, Pericardial Effusion; TAM, Transient Abnormal Myelopoiesis.

Kusanagi et al^9^ suggest that eosinophilic infiltration at the cardiac site may have caused direct heart damage and failure or that the degranulation of the eosinophils caused PE and consequent cardiomegaly. In the investigation of possible immunological bases for the development of effusion, Shitara et al^10^ did not observe a correlation between the increased levels of proinflammatory cytokines in the fluid when compared to the serum. The authors concluded that tumor necrosis factor‐alpha (TNF‐α) and interleukin‐6 (IL‐6) could be involved in fluid retention by increasing the vascular permeability, whereas interleukin‐13 (produced by Th2 lymphocytes) may have played a role in the increase and recruitment of eosinophils into the pericardial fluid. In addition, in agreement with Suda et al,^8^ Shenoy et al^5^ suggested that benign infiltration of the pericardium with eosinophils could be explained by the differentiation capacity of TAM blasts into the different cell lineages.

Most of the reported cases presented a benign course with no need for chemotherapy. In our case, due to clinical complications, low doses of chemotherapy were provided. Although in cases of TAM associated with DS, the presence of cavity effusion has been related to an increase in morbidity^5^, ^6^ and in this special case, the increased number of eosinophils/basophils in the pericardial fluid could have contributed to the worse course of disease.

In this rare case of TAM, a possible mechanism to explain the presence of a basophilic/eosinophilic PE without the increase in these cells in the PB is that cells from the PB could infiltrate the cardiac tissue and, due to a favorable microenvironment stimulated by the secretion of cytokines and growth factors, differentiate into basophils/eosinophils. Another point to highlight is that although the karyotype did not show any chromosomal change other than the trisomy 21, additional molecular tests were not performed. Mutations in the GATA1 gene are present in patients with DS and TAM, and the GATA gene family plays a pivotal role in the differentiation and maturation of multipotent progenitors into basophils/mast cells and eosinophils by encoding transcription factors and regulating their homeostasis.^4^, ^7^ Therefore, we can suppose that mutations in this gene may also have caused the differentiation of the blasts into eosinophils and basophils/mast cells under favorable microenvironment conditions.

## CONFLICT OF INTEREST

The authors stated they have no conflicts of interest.

## AUTHOR CONTRIBUTIONS

BFF: involved in data collection, study design, data analysis, and manuscript writing. BD, DKF, and CSF: involved in data collection and analysis and manuscript writing. DCBR: involved in data collection and manuscript writing. LA: involved in study design, data analysis, manuscript writing, and revision.
